# A two step synthesis of a key unit B precursor of cryptophycins by asymmetric hydrogenation

**DOI:** 10.3762/bjoc.7.32

**Published:** 2011-02-22

**Authors:** Benedikt Sammet, Mathilde Brax, Norbert Sewald

**Affiliations:** 1 Bielefeld University, Department of Chemistry, Organic and Bioorganic Chemistry, Universitätsstraße 25, 33615 Bielefeld, Germany

**Keywords:** amino acid, asymmetric hydrogenation, cryptophycin, DuPhos

## Abstract

A novel highly enantioselective two step access to a unit B precursor of cryptophycins in good yields from commercially available starting materials has been developed. The key step is an asymmetric hydrogenation using the commercially available [(COD)Rh-(*R*,*R*)-Et-DuPhos]BF_4_ catalyst. The synthetic route provides the advantage of less synthetic steps, proceeds with high yields and enantioselectivity, and avoids hazardous reaction conditions.

## Introduction

**Figure 1:**
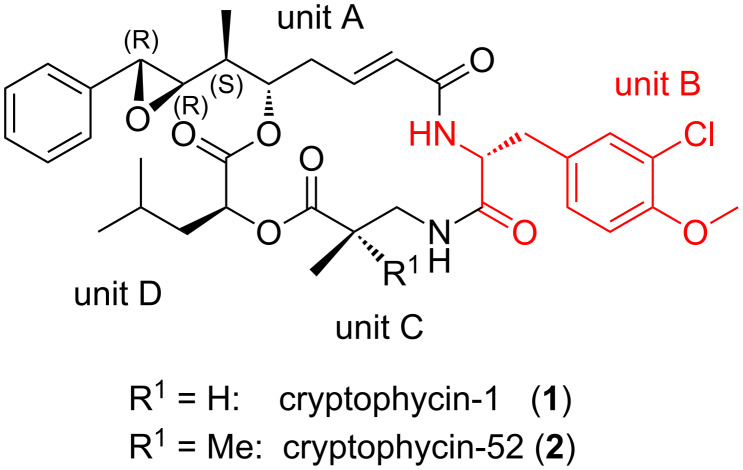
The four building blocks (units) A–D of cryptophycin-1 (**1**) and cryptophycin-52 (**2**).

Cryptophycins are macrocyclic depsipeptides, which show very high cytotoxicity even against multidrug-resistant cell lines. They inhibit mitosis of eukaryotic cells by interacting with the β-subunit of α/β-tubulin heterodimers. Numerous natural and artificial analogs have been analysed in structure–activity relationship (SAR) studies. The unit B of cryptophycins contains a considerably modified D-tyrosine derivative (Figure 1). Substituent variations at unit B are not well tolerated. Both the methoxy and the chloro substituent are required for full biological activity [1–4].

**Scheme 1:**
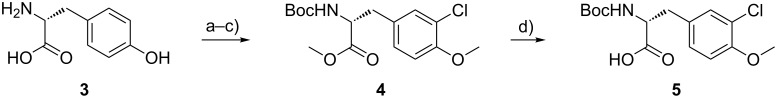
Synthesis of the unit B precursor from D-tyrosine (**3**). Reagents and conditions [7]: a) SO_2_Cl_2_, AcOH, rt, 90 min, (75%); b) Boc_2_O, NaOH, *t*-BuOH/H_2_O, rt, 16 h (quant.) c) Me_2_SO_4_, K_2_CO_3_, acetone, reflux, 4 h (99%); d) LiOH, H_2_O/THF/MeOH, rt, 1 h (93%).

The previously published synthetic route to unit B precursor **4** involves a three-step modification of D-tyrosine by chlorination, protecting group introduction and double methylation followed by a final saponification reaction to give carboxylic acid **5** (Scheme 1). A number of experimental procedures for this route have been published [5–7]. The selective monochlorination of D-tyrosine is quite cumbersome since the formation of the dichlorinated product must be minimized and the presence of unreacted D-tyrosine after the reaction must be completely avoided. The dichlorinated by-product has to be separated by column chromatography when purifying the desired methyl ester **4** [7–8]. In addition, another major drawback of this synthetic route is the use of highly toxic and carcinogenic dimethyl sulfate.

**Scheme 2:**
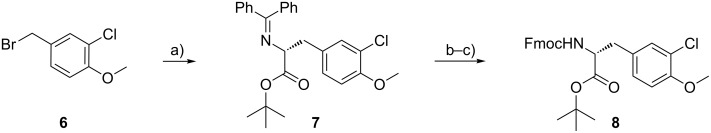
Unit B synthesis by a chiral PTC approach. Reagents and conditions [9]: a) *N*-(Diphenylmethylene)glycine *tert*-butyl ester, 50% KOH, toluene/CHCl_3_, chiral phase transfer catalyst (0.01 equiv), 0 °C, 20 h (87%; 96% *ee*); b) 15% citric acid, THF, rt, 16 h; c) FmocCl, Na_2_CO_3_, THF, rt, 14 h, (72% over two steps).

A completely different route to unit B precursor **8** (Scheme 2) is based on a phase transfer catalyst (PTC) mediated asymmetric alkylation. However, the required cinchonine derived chiral catalyst is not commercially available [9].

## Results and Discussion

**Scheme 3:**
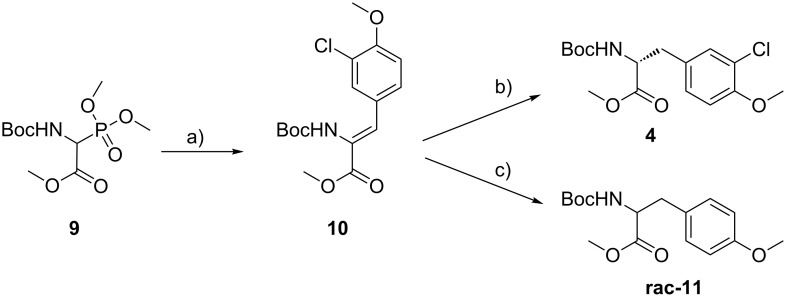
Unit B precursor **4** synthesis by asymmetric hydrogenation. Reagents and conditions: a) 3-Chloro-4-methoxybenzaldehyde, 1,1,3,3-tetramethylguanidine, CH_2_Cl_2_, 0 °C to rt, 16 h (84%); b) [(COD)Rh-(*R*,*R*)-Et-DuPhos]BF_4_ (1.9 mol %), H_2_, dry and degassed MeOH, 3–6 bar, 21.5 h (97%; 98% *ee*); c) 10% Pd/C, H_2_, MeOH, 16 h, (76%).

We envisaged a two step synthesis for the unit B precursor **4** (Scheme 1) from commercially available non-toxic starting materials based on an asymmetric hydrogenation approach to make the unit B precursor synthesis shorter and safer. In general, there is also a whole variety of possible stereoselective synthetic methods available to synthesize modified α-amino acids, such as the classical Schöllkopf-method [10] or catalytic approaches [11–12]. The unit B precursor of cryptophycin is a phenylalanine derivative. An asymmetric hydrogenation approach for the synthesis of such α-amino acids is well-established [12]. In the first step of the developed synthesis 3-chloro-4-methoxybenzaldehyde is reacted with *rac*-Boc-α-phosphonoglycine trimethyl ester (**9**) [13–14] to yield olefin **10** in a completely *Z*-selective Horner–Wadsworth–Emmons (HWE) reaction (Scheme 3). Asymmetric hydrogenation using the commercially available [(COD)Rh-(*R*,*R*)-Et-DuPhos]BF_4_ catalyst [14–15] gave the anticipated methyl ester **4** (Scheme 1) in 97% yield with an *ee* exceeding 98% (determined by chiral HPLC). Hydrogenation of **10** with 10% Pd/C was envisaged to obtain ***rac***-**4** as a reference for the determination of the *ee*. Interestingly, due to this more reactive catalyst a complete reductive dehalogenation was observed to give *rac*-Boc-Tyr(Me)-OMe (***rac*-11**) as reported for a similar case [16]. Therefore, ***ent***-**4** was synthesized analogously also using the commercially available enantiomeric catalyst ([(COD)Rh-(*S*,*S*)-Et-DuPhos]BF_4_).

## Conclusion

A novel two step synthesis of the important cryptophycin unit B precursor **4** is disclosed based on a HWE reaction followed by a highly enantioselective [(COD)Rh-(*R*,*R*)-Et-DuPhos]BF_4_ mediated asymmetric hydrogenation. This high-yielding access is more convenient and avoids hazardous chemicals in contrast to the established method.

## Supporting Information

File 1 Full experimental procedures and detailed analytical data for the synthesis of **10** and **4** including chiral HPLC spectra.

## References

[1] Trimurtulu G, Ohtani I, Patterson G M L, Moore R E, Corbett T H, Valeriote F A, Demchik L (1994). J Am Chem Soc.

[2] Eißler S, Stoncius A, Nahrwold M, Sewald N (2006). Synthesis.

[3] Nahrwold M, Bogner T, Eissler S, Verma S, Sewald N (2010). Org Lett.

[4] Sammet B, Bogner T, Nahrwold M, Weiss C, Sewald N (2010). J Org Chem.

[5] Barrow R A, Hemscheidt T, Liang J, Paik S, Moore R E, Tius M A (1995). J Am Chem Soc.

[6] McCubbin J A, Maddess M L, Lautens M (2006). Org Lett.

[7] Nahrwold M (2009). β2-Aminosäuren als Bausteine funktionalisierter Cryptophycin-Analoga.

[8] Eißler S (2008). Synthese von Cryptophycinen für SAR-Studien.

[9] Danner P, Bauer M, Phukan P, Maier M E (2005). Eur J Org Chem.

[10] Lim H J, Gallucci J C, RajanBabu T V (2010). Org Lett.

[11] Zuend S J, Coughlin M P, Lalonde M P, Jacobsen E N (2009). Nature.

[12] Nájera C, Sansano J M (2007). Chem Rev.

[13] Schmidt U, Griesser H, Leitenberger V, Lieberknecht A, Mangold R, Meyer R, Riedl B (1992). Synthesis.

[14] Bower J F, Szeto P, Gallagher T (2005). Chem Commun.

[15] Burk M J, Feaster J E, Nugent W A, Harlow R L (1993). J Am Chem Soc.

[16] Melillo D G, Larsen R D, Mathre D J, Shukis W F, Wood A W, Colleluori J R (1987). J Org Chem.

