# Supplementary material for: A two step synthesis of a key unit B precursor of cryptophycins by asymmetric hydrogenation
Source: Beilstein J Org Chem. 2011 Feb 22;7:243–5. doi: 10.3762/bjoc.7.32 (PMC3063055; doi:10.3762/bjoc.7.32)
Supplement: File 1 — Full experimental procedures and detailed analytical data for the synthesis of 10 and 4 including chiral HPLC spectra. [file Beilstein_J_Org_Chem-07-243-s001.pdf]

Supporting Information

for

A two step synthesis of a key unit B precursor of cryptophycins by asymmetric hydrogenation

Benedikt Sammet, Mathilde Brax and Norbert Sewald*

Address: Bielefeld University, Department of Chemistry, Organic and Bioorganic Chemistry, Universitätsstraße 25, 33615 Bielefeld, Germany

Email: Norbert Sewald - norbert.sewald@uni-bielefeld.de

* Corresponding author

Full experimental procedures and detailed analytical data for the synthesis of 10 and 4 including chiral HPLC spectra.

General information

CH_2Cl_2 was dried over CaH_2. Dry methanol was obtained by carefully adding magnesium shavings and a few iodine crystals to an excess of methanol (Caution!) and subsequent heating to reflux. TLC plates were stained by dipping into ammonium heptamolybdate/cerium(IV) sulfate solution [10.0 g $(NH_4)_6Mo_7O_{24} \cdot 4\ H_2O$, 0.4 g $Ce(SO_4)_2$, 5.4 mL conc. H_2SO_4, 180 mL H_2O], followed by subsequent heating. All NMR spectra were recorded at 298 K in $CDCl_3$. TMS was used for internal calibration (^{1}H NMR and ^{13}C NMR: 0.00 ppm). IR spectra were obtained on an instrument containing an ATR accessory. The enantiomeric excess (*ee*) of the asymmetric hydrogenation reaction utilizing [(COD)Rh-(*R*,*R*)-Et-DuPhos]BF_4 and its enantiomer for comparison was determined by chiral HPLC (Chiralpak AD, hexane/2-propanol 9:1, 1 mL/min, 254 nm).

Experimental

(*Z*)-Methyl 3-(3-chloro-4-methoxyphenyl)-2-(*tert*-butoxycarbonylamino)acrylate (10):

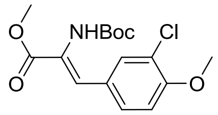

10

According to a slightly modified literature procedure [1] 3-chloro-4-methoxybenzaldehyde (0.449 g, 2.63 mmol) and *rac*-Boc-α-phosphonoglycine trimethyl ester (**9**) (1.00 g, 3.36 mmol) were dissolved in dry CH_2Cl_2 (26 mL) at 0 °C. 1,1,3,3-Tetramethylguanidine (0.496 mL, 0.455 g, 3.95 mmol) was added over a 15 min period. After 4 h at 0 °C the solution was stirred overnight at rt. CH_2Cl_2 (100

mL) was added and the organic phase washed with 10% tartaric acid (40 mL), then brine (20 mL) and dried over solid $MgSO_4$. The solvent was removed under reduced pressure. Purification by column chromatography (hexane/EtOAc: 3:1) gave olefin **10** (0.761 g, 84%) as a colorless solid. R_f (hexane/EtOAc: 7:3) = 0.47; **^{1}H NMR** (300 MHz, $CDCl_3$): δ [ppm] = 1.43 (s, 9H, $NHCO_2C(C\underline{H}_3)_3$), 3.85 (s, 3H, $CO_2C\underline{H}_3$), 3.92 (s, 3H, $C_{ar}OC\underline{H}_3$), 6.24 (bs, 1H, N$\underline{H}$Boc), 6.90 (d, J = 8.6 Hz, 1H, $C^{5'}\underline{H}$), 7.22 (s, 1H, $CO_2C{=}C\underline{H}$), 7.40 (dd, J = 1.5 Hz, 8.5 Hz, 1H, $C^{6'}\underline{H}$) 7.64 (d, J = 2.1 Hz, 1H, $C^{2'}\underline{H}$). **^{13}C NMR** (151 MHz, $CDCl_3$) δ [ppm] = 28.2 ($NHCO_2C(\underline{C}H_3)_3$), 52.7 ($CO_2\underline{C}H_3$), 56.2 ($C_{ar}O\underline{C}H_3$), 81.1 ($\underline{C}(CH_3)_3$), 111.5 ($\underline{C}^{5'}H$), 122.5 ($CO_2\underline{C}{=}CH$), 127.7 ($\underline{C}^{1'}$ and $\underline{C}^{3'}Cl$), 129.1 ($CO_2C{=}\underline{C}H$), 130.3 ($\underline{C}^{6'}H$), 131.3 ($\underline{C}^{2'}H$), 155.5 ($NH\underline{C}O_2C(CH_3)_3$ and $\underline{C}^{4'}OCH_3$), 166.1 ($\underline{C}O_2CH_3$). **ESI–MS:** *m/z* 363.9 $[M+Na]^+$. **HR–ESI:** calculated for $C_{16}H_{20}N_1O_5ClNa$ $[M+Na]^+$ 364.09222, found 364.09215. **IR** (neat, cm^{-1}): 3211, 3103, 1697, 1504, 804. **Elemental analysis:** calculated (%) for $C_{16}H_{20}ClNO_5$: C 56.32, H 5.90, N 4.10; found: C 56.31, H 6.04, N 4.07.

(*R*)-Methyl 3-(3-chloro-4-methoxyphenyl)-2-(*tert*-butoxycarbonylamino)propanoate (4):

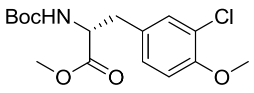

4

According to a slightly modified literature procedure [1] dry and degassed methanol (16 mL) was added to olefin **10** (0.410 g, 1.20 mmol) and [(COD)Rh-(*R*,*R*)-Et-DuPhos]BF_4 (0.015 g, 0.023 mmol, 1.9 mol %) in a hydrogenation flask. After three purging cycles the initial suspension was stirred under a hydrogen atmosphere (3–6 bar) for 21.5 h in a hydrogenation apparatus. The solvent was removed in vacuo and the catalyst separated from the already highly pure product by column chromatography (hexane/EtOAc: 3:1) to yield ester **4** (0.399 g, 97%, 98% *ee*) as a

colorless oil. $\boldsymbol{R_f}$ (hexane/EtOAc: 7:3) = 0.63; HPLC (Chiralpak AD, hexane/2-propanol 9:1, 1 mL/min, 254 nm): t_R = 9.8 min (**4**), 11.2 min (***ent*-4**). $[\alpha]_D^{24}$ = −49 (c = 0.78 in $CHCl_3$; Lit. $[\alpha]_D^{24}$ = −45 [2]). **^{1}H NMR** (300 MHz, $CDCl_3$): δ [ppm] = 1.43 (s, 9H, $NHCO_2C(C\underline{H}_3)_3$), 2.96 (dd, J = 5.9, 14.1 Hz, 1H, $C\underline{H}^AH^B$), 3.06 (dd, J = 5.6 Hz, 14.0 Hz, 1H, $CH^A\underline{H}^B$), 3.73 (s, 3H, $CO_2C\underline{H}_3$), 3.88 (s, 3H, $C_{ar}OC\underline{H}_3$), 4.52 (m, 1H, $C^{\alpha}\underline{H}$), 4.99 (d, J = 7.5 Hz, 1H, $N\underline{H}$), 6.85 (d, J = 8.4 Hz, 1H, $C^{5'}\underline{H}$), 6.99 (dd, J = 2.1 Hz, 8.4 Hz, 1H, $C^{6'}\underline{H}$), 7.13 (d, J = 1.6 Hz, 1H, $C^{2'}\underline{H}$). **^{13}C NMR** (151 MHz, $CDCl_3$) δ [ppm] = 28.3 ($NHCO_2C(\underline{C}H_3)_3$), 37.2 ($\underline{C}H^AH^B$), 52.3 ($CO_2\underline{C}H_3$), 54.4 ($\underline{C}^{\alpha}H$), 56.1 ($C^{4'}O\underline{C}H_3$), 80.1 ($\underline{C}(CH_3)_3$), 112.1 ($\underline{C}^{5'}H$), 122.3 ($\underline{C}^{3'}Cl$), 128.5 ($\underline{C}^{6'}H$), 129.1 ($\underline{C}^{1'}$), 131.1 ($\underline{C}^{2'}H$), 154.1 ($\underline{C}^{4'}OCH_3$), 155.0 ($NH\underline{C}O_2C(CH_3)_3$), 172.1 ($\underline{C}O_2CH_3$).

The ^{1}H and ^{13}C NMR data are in complete agreement with previously published data, where **4** was synthesized by the chlorination and methylation of D-tyrosine [3]. However, the ^{13}C NMR shift value of the aromatic carbon $C^{3'}Cl$ (122.3 ppm), where the chloro substituent is attached to, disagrees with specifications from two other references: 135.6 ppm [2] and 132.4 ppm [4]. As we have obtained compound **4** according to a completely different route, we are confident that our data is correct.

ESI–MS: *m/z* 366.0 [M+Na]$^+$; 708.6 [2M+Na]$^+$.

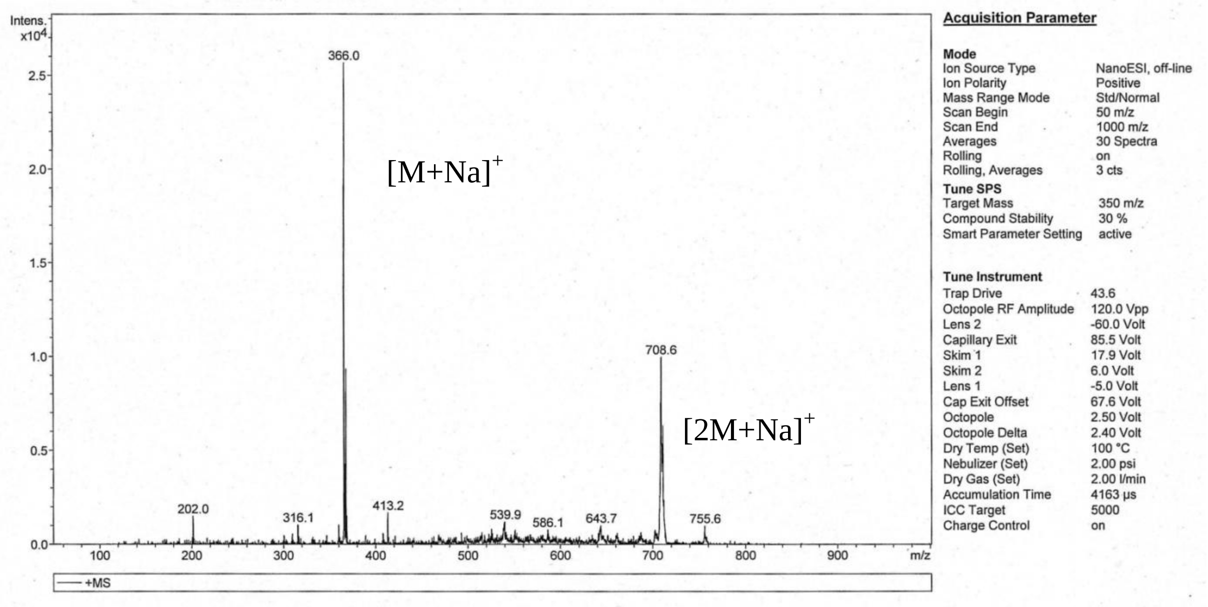

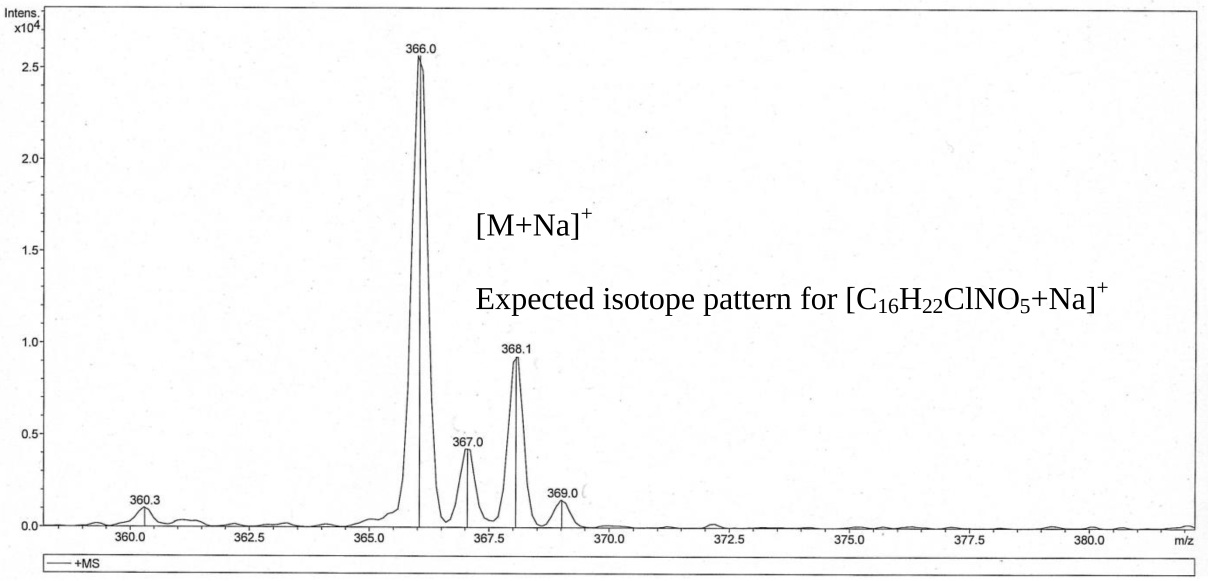

Elemental analysis: calculated (%) for $C_{16}H_{22}ClNO_5$: C 55.90, H 6.45, N 4.07; found: C 55.74, H 6.31, N 4.00.

^{1}H NMR: (*Z*)-Methyl 3-(3-chloro-4-methoxyphenyl)-2-(*tert*-butoxycarbonylamino) acrylate (10)

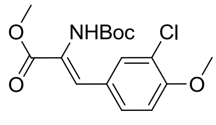

10

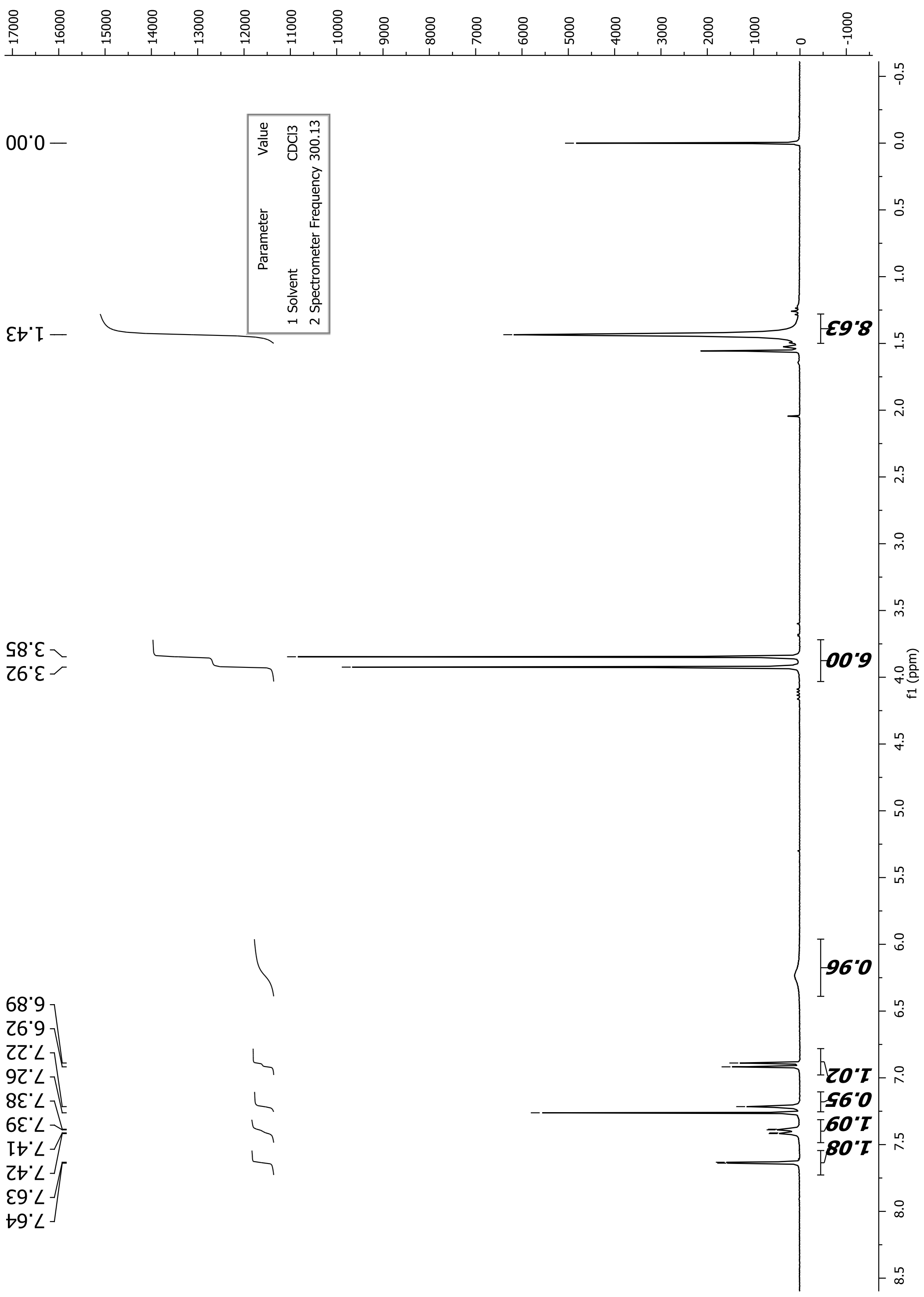

^{13}C NMR: (*Z*)-Methyl 3-(3-chloro-4-methoxyphenyl)-2-(*tert*-butoxycarbonylamino) acrylate (10)

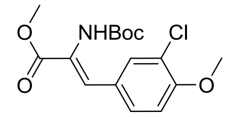

10

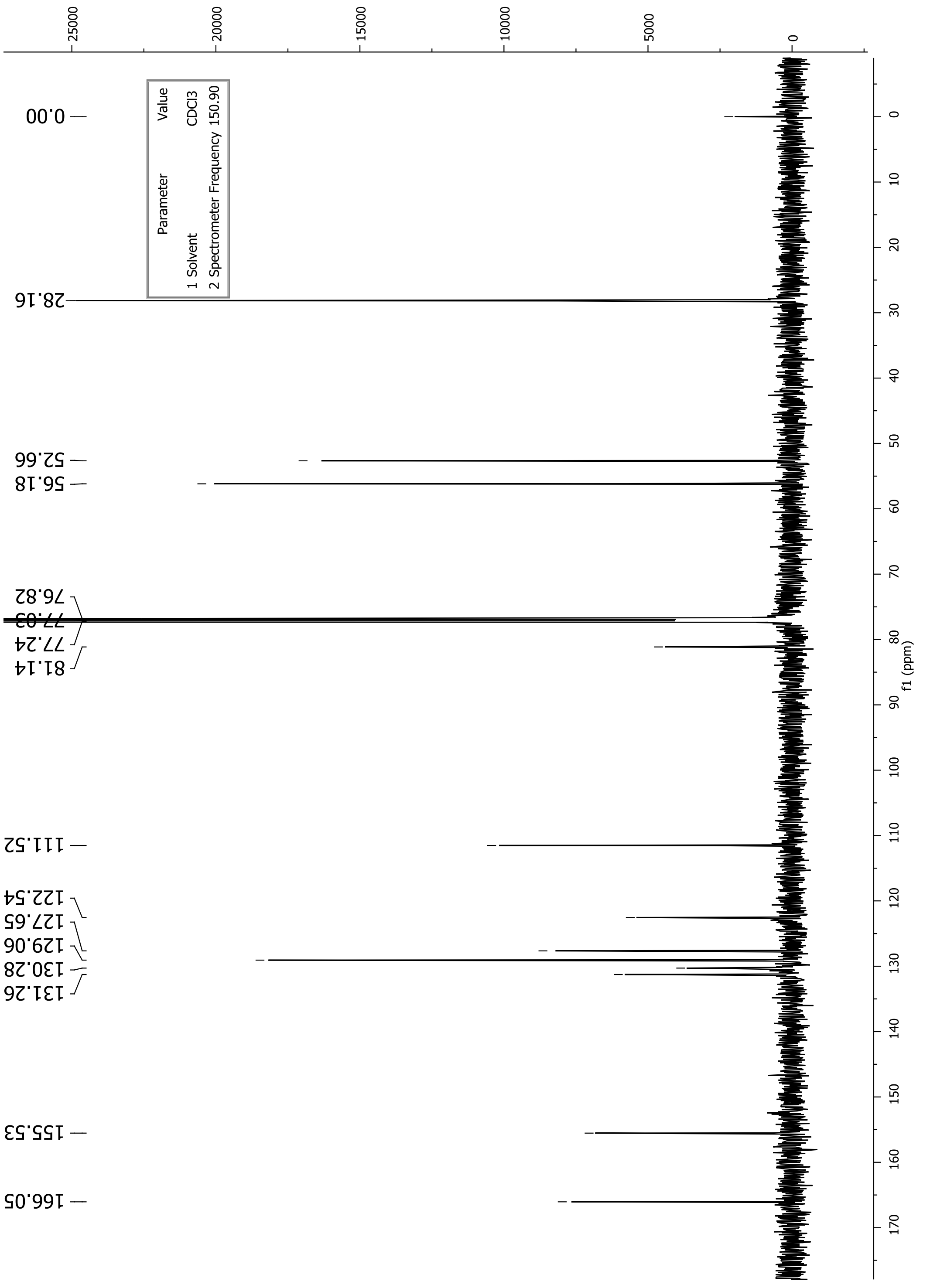

^{1}H NMR: (*R*)-Methyl 3-(3-chloro-4-methoxyphenyl)-2-(*tert*-butoxycarbonyl-amino)propanoate (4)

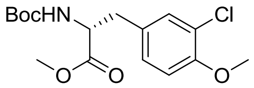

4

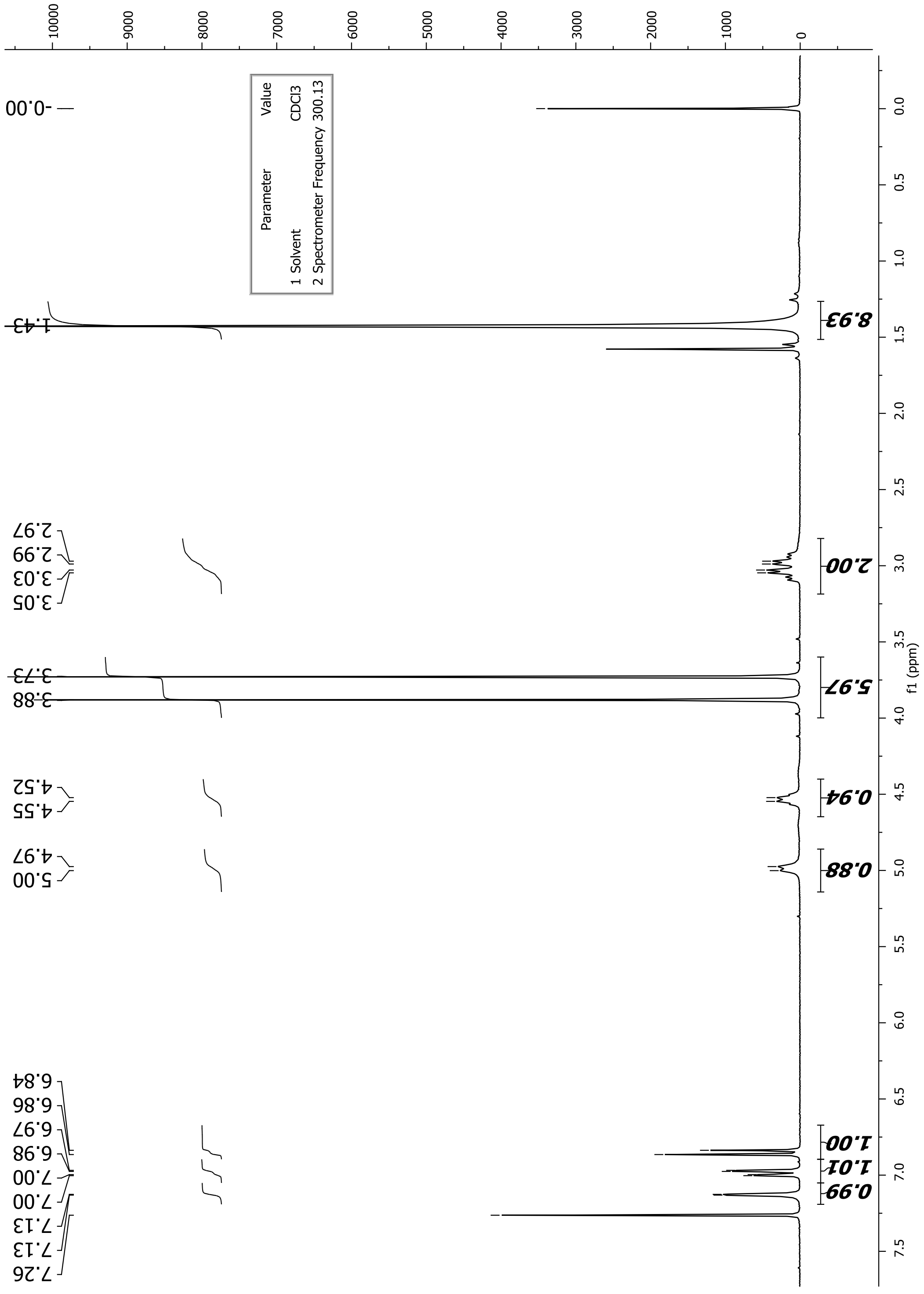

^{13}C NMR: (*R*)-Methyl 3-(3-chloro-4-methoxyphenyl)-2-(*tert*-butoxycarbonyl-amino)propanoate (4)

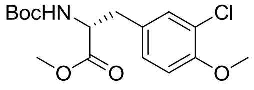

4

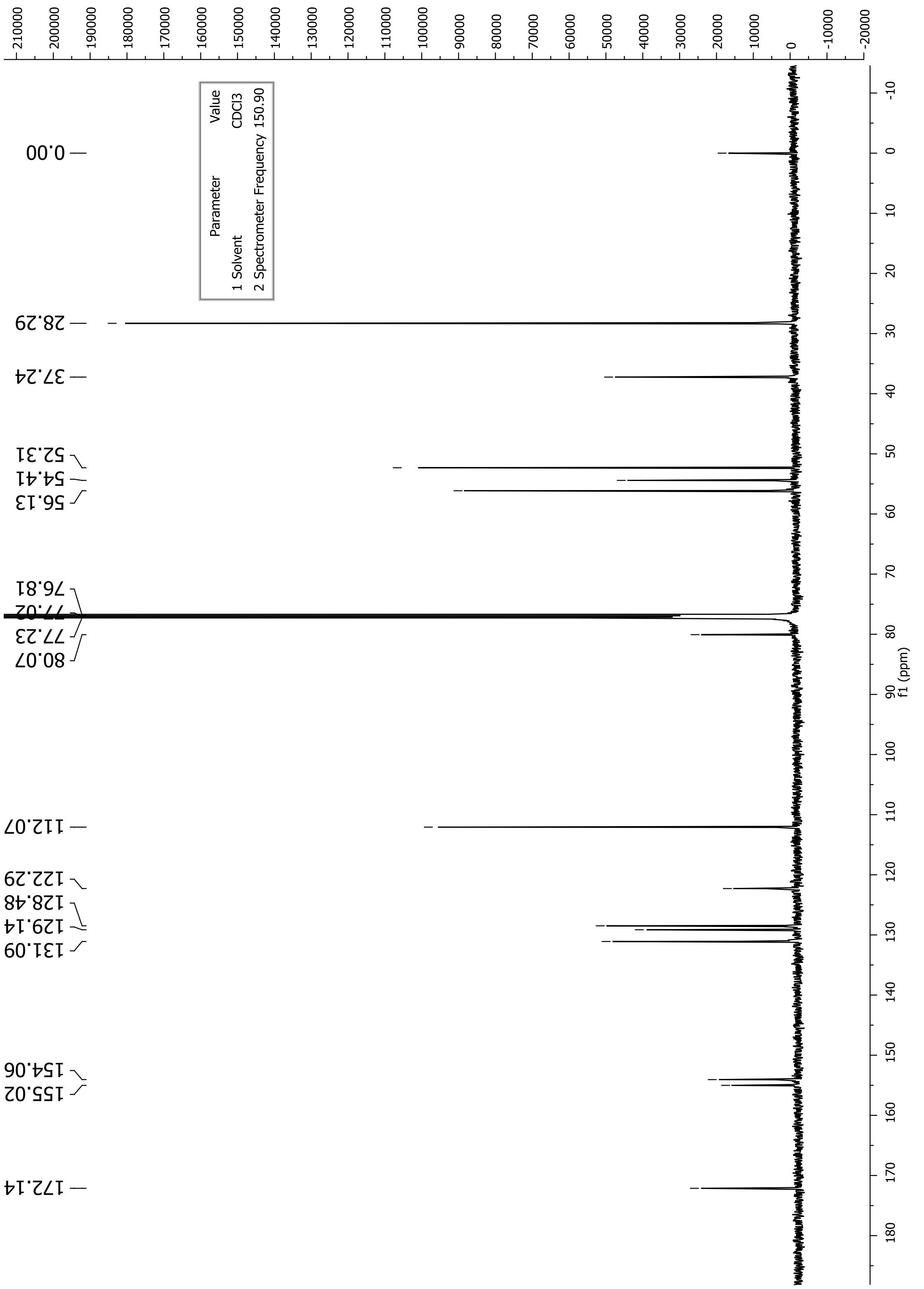

Chiral HPLC Run: Synthesis of (*R*)-methyl 3-(3-chloro-4-methoxyphenyl)-2-(*tert*-butoxycarbonylamino)propanoate (4)

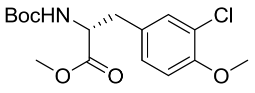

4 t_R = ca. 9.8 min (**4**)

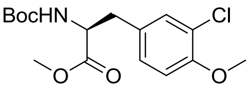

ent-4 t_R = ca.11.2 min (***ent*-4**)

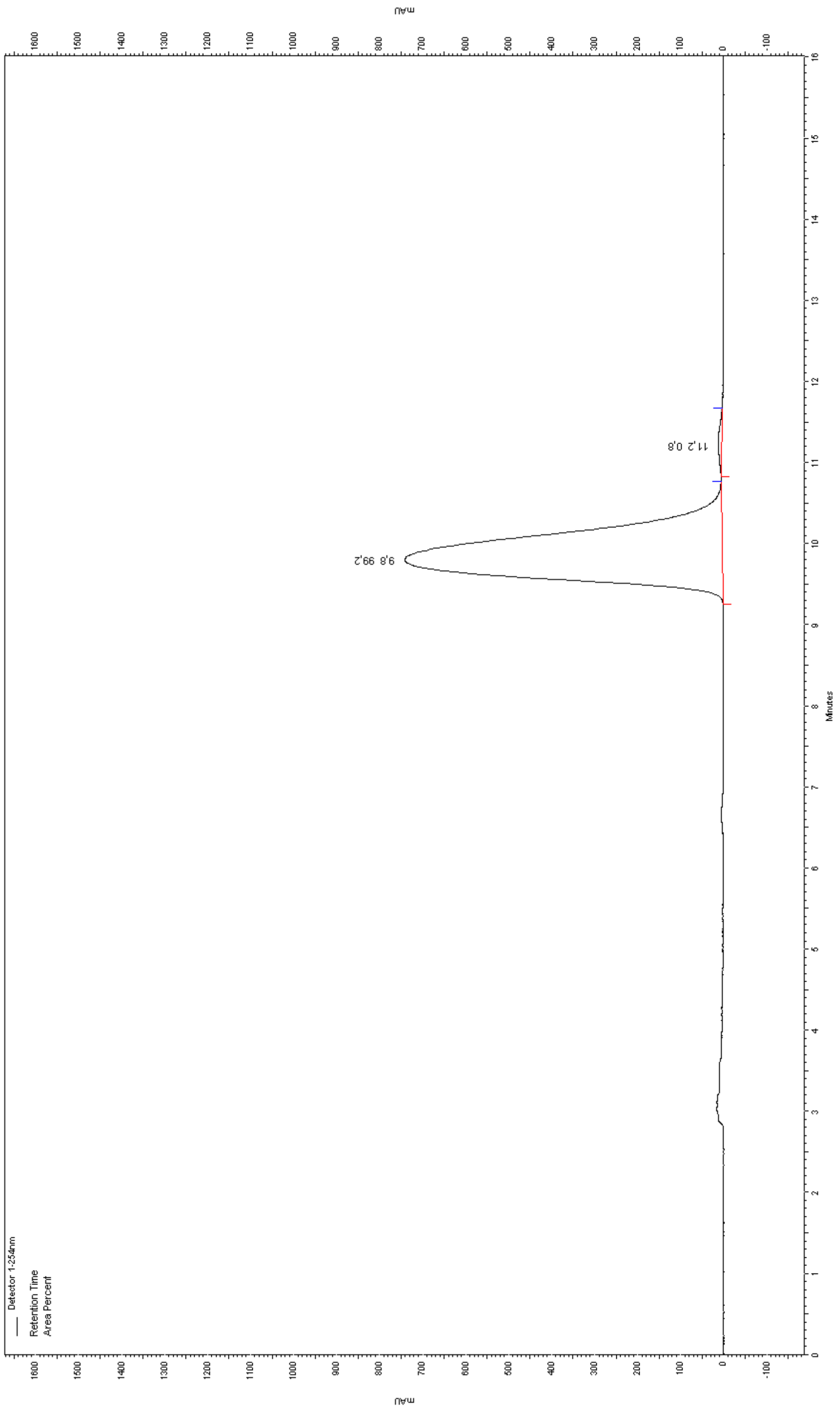

Chiral HPLC Run: Synthesis of (*S*)-methyl 3-(3-chloro-4-methoxyphenyl)-2-(*tert*-butoxycarbonylamino)propanoate (*ent*-4)

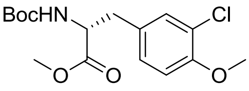

4

t_R = ca. 9.8 min (**4**)

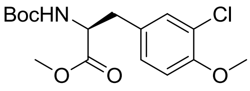

ent-4

t_R = ca.11.2 min (***ent*-4**)

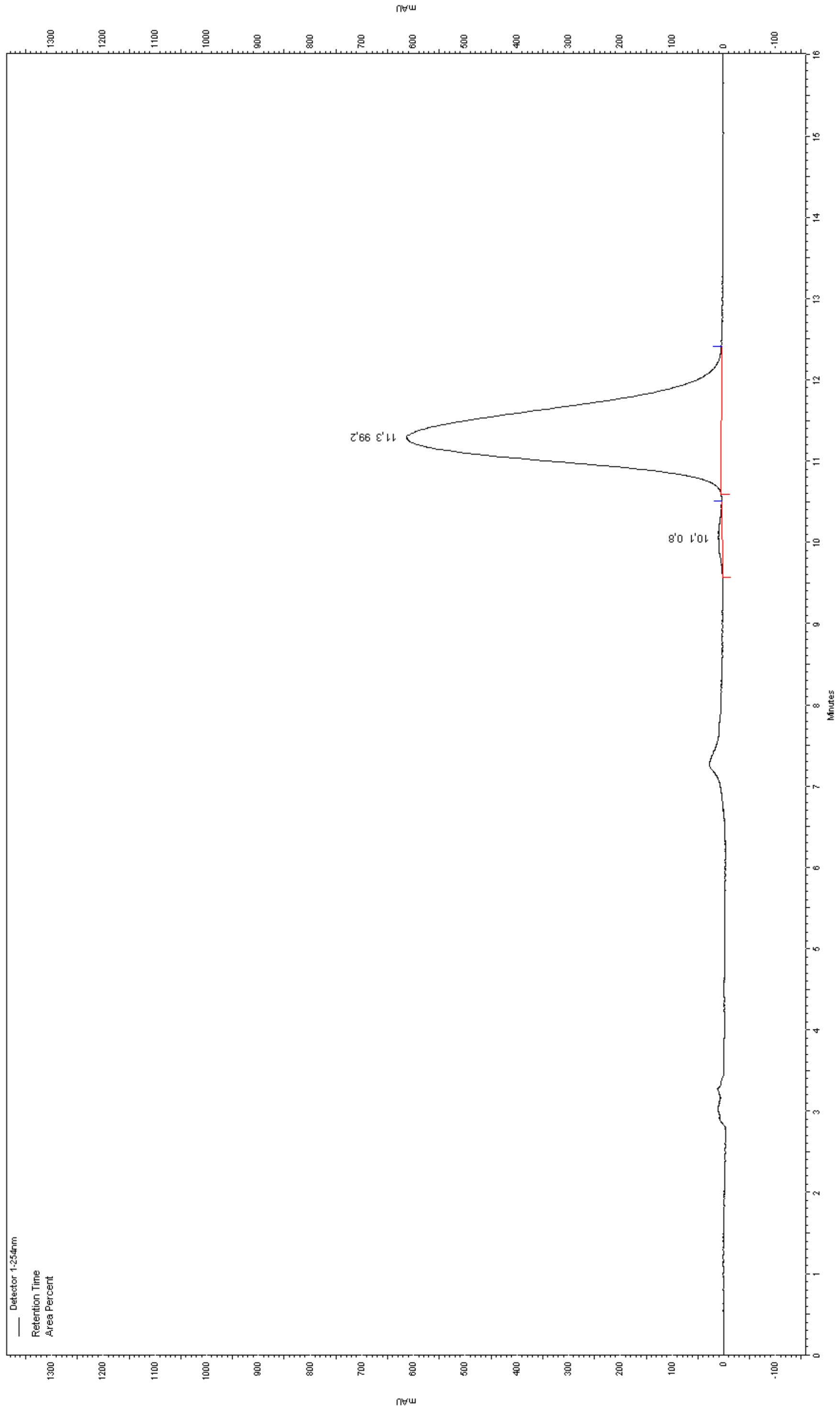

Chiral HPLC Run: Mixture of 4 and *ent*-4

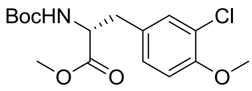

4 t_R = ca. 9.8 min (**4**)

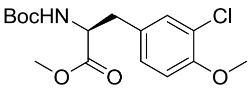

ent-4 t_R = ca.11.2 min (***ent*-4**)

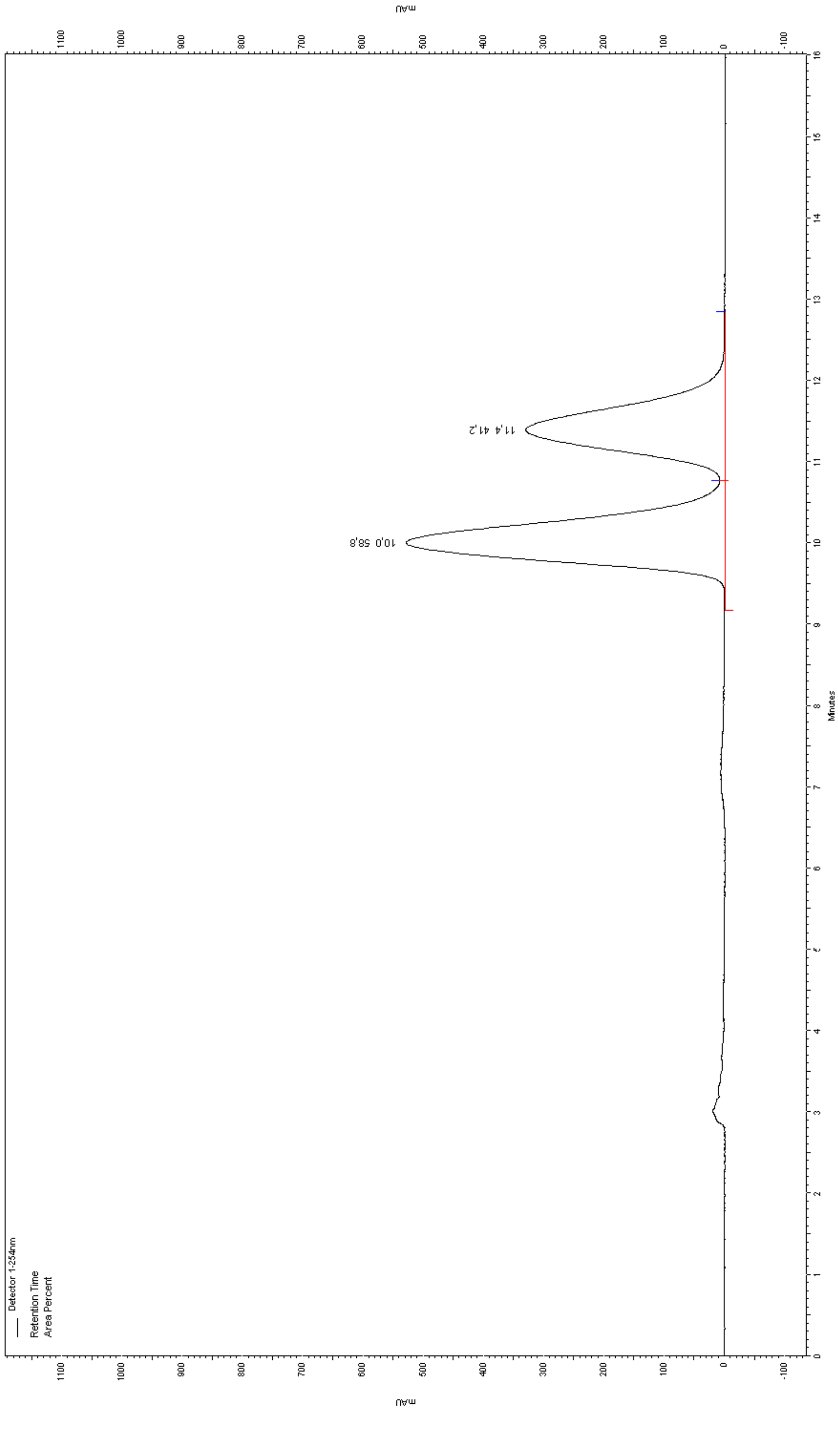

Literature

[1] Bower, J. F.; Szeto, P.; Gallagher, T. *Chem. Comm.* **2005**, 5793-5795.

[2] Barrow, R. A.; Hemscheidt, T.; Liang, J.; Paik, S.; Moore, R. E.; Tius, M. A. *J. Am. Chem. Soc.* **1995**, *117*, 2479-2490

[3] Nahrwold, M. β^2-Aminosäuren als Bausteine funktionalisierter Cryptophycin-Analoga, Ph.D. Thesis, Bielefeld University, Germany, **2009**. http://bieson.ub.uni-bielefeld.de/volltexte/2010/1673/ (accessed Dec 19, **2010**).

[4] McCubbin, J. A.; Maddess, M. L.; Lautens, M. *Org. Lett.* **2006**, *8*, 2993-2996.
